# Supplementary material for: The thylakoid proton antiporter KEA3 regulates photosynthesis in response to the chloroplast energy status
Source: Nat Commun. 2024 Mar 30;15:2792. doi: 10.1038/s41467-024-47151-5 (PMC10981695; doi:10.1038/s41467-024-47151-5)
Supplement: Supplementary file 1 — Supplementary Information [file 41467_2024_47151_MOESM1_ESM.pdf]

The thylakoid proton antiporter KEA3 regulates photosynthesis in response to the chloroplast energy status

Michał Uflewski[1#], Tobias Rindfleisch[1,2,3#], Kübra Korkmaz[1], Enrico Tietz[1], Sarah Mielke[1], Viviana Correa Galvis[1], Beatrix Dünschede[4], Marcin Luzarowski[1], Aleksandra Skirycz[1], Markus Schwarzländer[5], Deserah D. Strand[1], Alexander P. Hertle[1], Danja Schünemann[4], Dirk Walther[1], Anja Thalhammer[2], Martin Wolff[2] and Ute Armbruster[1,6,7*]

Supplementary Figures 1-13

Supplementary Tables 1-3

Supplementary References

Supplementary Figures

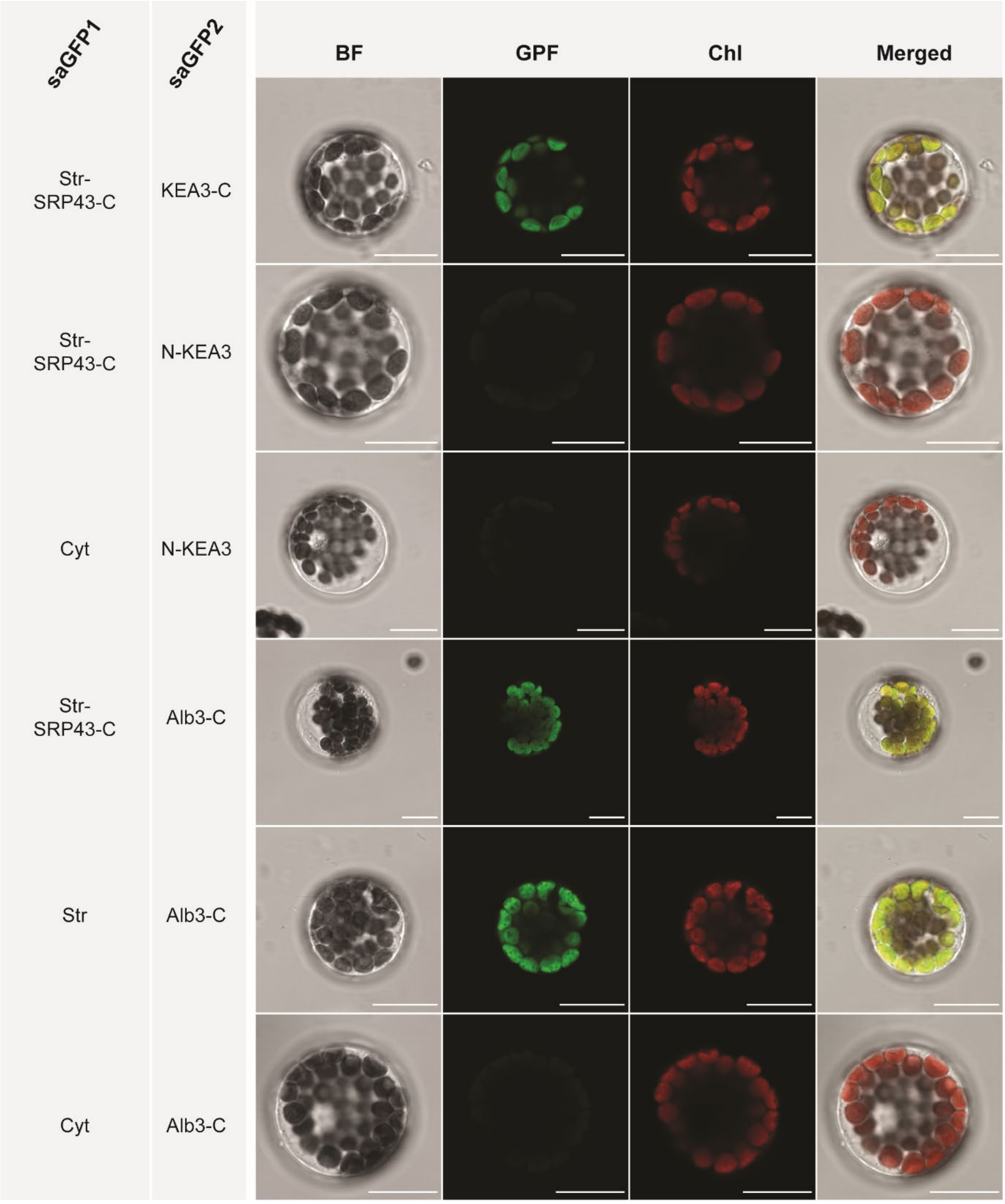

Supplementary Fig. 1 Controls for the self-assembly GFP analysis. The larger self-assembly GFP fragment 1 (saGFP1) was either targeted to the stroma by a chloroplast transit peptide (Str) or as a fusion with precursor cpSRP43 (Str-SRP43-C). The smaller saGFP fragment 2 (saGFP2) was fused to the C-terminus of KEA3 (KEA3-C) or Alb3 (Alb3-C). Additionally, it was inserted between the chloroplast targeting peptide and the mature KEA3 yielding a chloroplast targeted saGFP2-N-KEA3 fusion. Arabidopsis protoplasts were co-transformed with saGFP1 and saGFP2 constructs. Only those protoplasts that were co-transformed with C-terminal fusions of saGFP2 to KEA3 or Alb3 and stromal localized saGFP1 (transit peptide alone or cpSRP43 fusion), yielded strong GFP fluorescence. Scale bar, 15 µm.

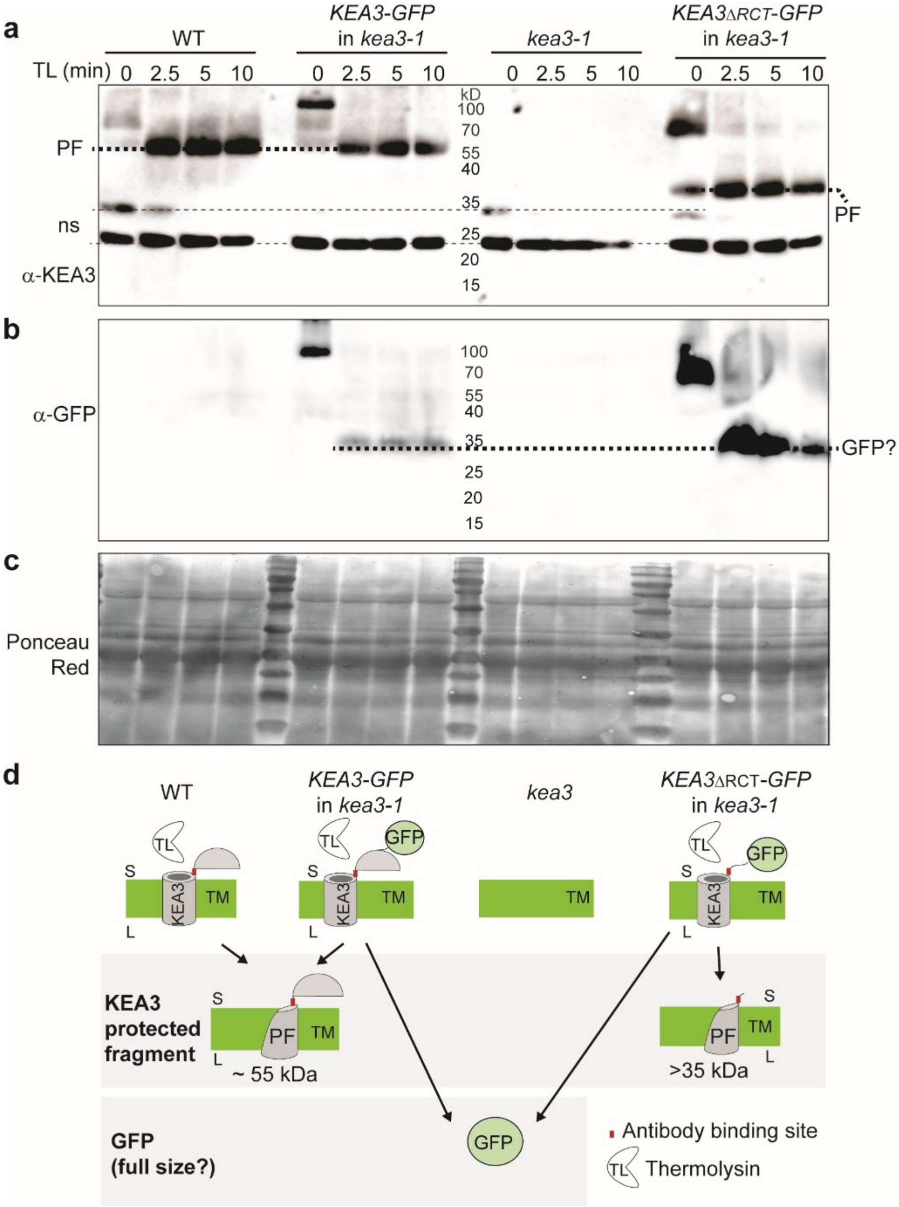

Supplementary Fig. 2. Thermolysin digestion of intact thylakoids supports a stromal localization of the RCT. **a-b**, Intact thylakoids from WT, *KEA3-GFP* in *kea3-1*, *kea3-1* and *KEA3$_{\Delta RCT}$-GFP* in *kea3-1* were treated with thermolysin and samples were taken at 0, 2.5, 5 and 10 min after addition of the protease. Proteins were separated by SDS-PAGE and detected by immunoblotting using a KEA3 (a) or GFP-specific (b) antibody. **c**, Ponceau Red staining of the membrane after blotting shows no difference between the different lengths of protease treatments. **d**, Model of a stromal localized RCT in line with the results shown in a-b. Thylakoids harboring either the native KEA3 or KEA3-eGFP showed a protected fragment of the same molecular mass after the thermolysin treatment, in line with a stromal exposed RCT being cut off by thermolysin. Thylakoids harboring a KEA3$_{\Delta RCT}$-eGFP version showed a protected smaller fragment, suggesting that the protected fragment in KEA3-eGFP comprises at least part of the C-terminus. Detection of eGFP showed fragments of a size corresponding to full-length eGFP, for both KEA3-eGFP and KEA3$_{\Delta RCT}$-eGFP after digestion. This may be due to the release of stromal eGFP from intact thylakoids by the thermolysin treatment, which would assume eGFP to be resistant to further fragmentation by thermolysin in the reaction mix. Alternatively, the fragment detected by the GFP antibody may represent two different hybrids of similar size consisting of part of GFP and fragments of either KEA3 or KEA3$_{\Delta RCT}$, respectively. (TL, thermolysin; PF, protected fragment; TM, thylakoid membrane; S, stroma and L, lumen, ns, nonspecific signal).

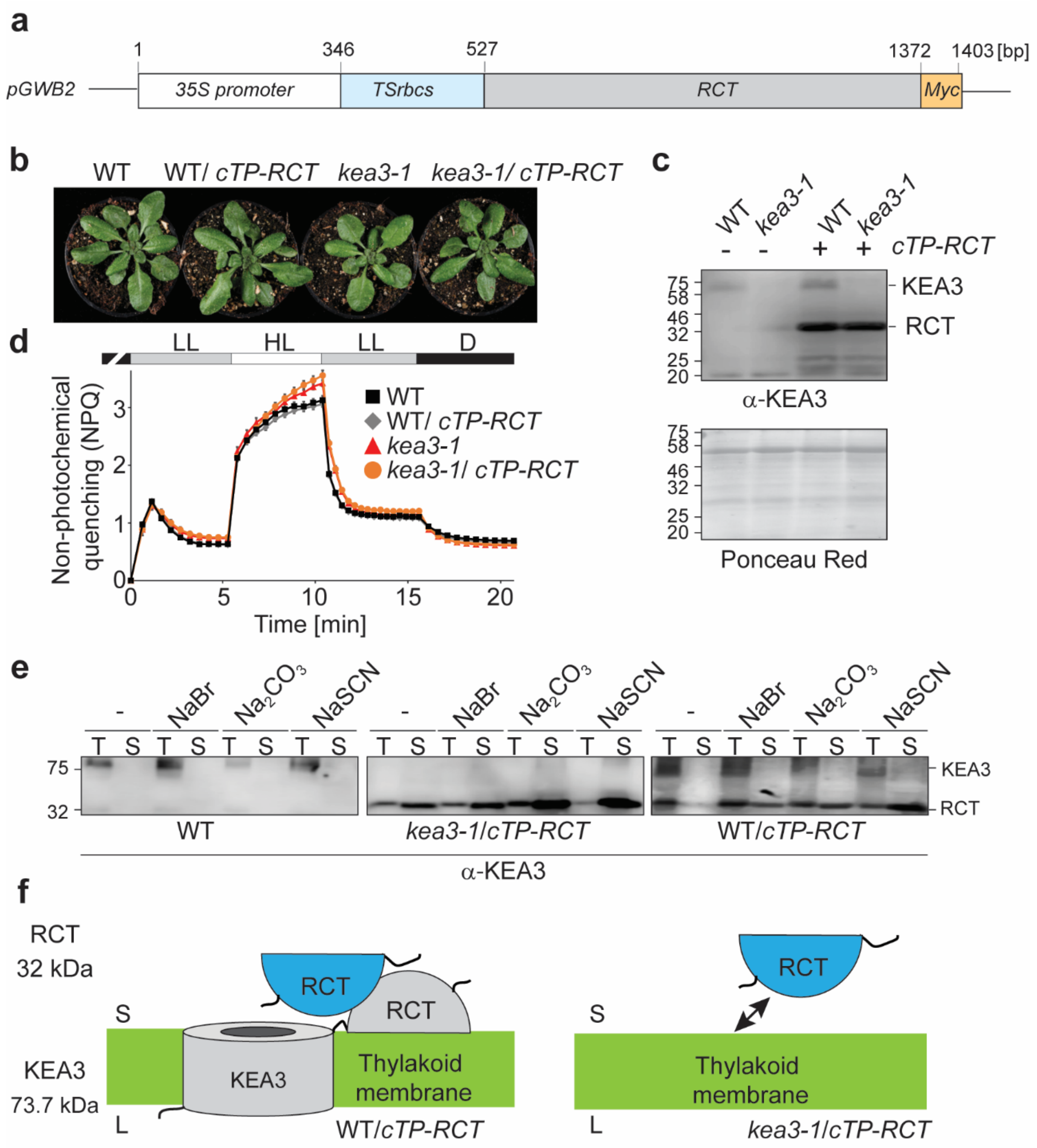

Supplementary Fig. 3. Native KEA3 stabilizes stromal RCT. a, Construct that targets the Myc-tagged KEA3 regulatory C-terminus (RCT, AAs 495-776) to the chloroplast stroma (*TSrbcs*, sequence for chloroplast targeting of Arabidopsis RbcS3B; *pGWB2*, binary vector used for cloning). **b**, Picture of four-week-old WT, *kea3-1* and *cTP-RCT* expressing plants. **c**, Immunoblot analysis of total protein extract from plants shown in b, using a KEA3 specific antibody, reveals *cTP-RCT* plants to accumulate high levels of additional RCT. The Ponceau Red stained membrane prior to detection is shown as a loading control. **d**, Non-photochemical quenching (NPQ) analysis of 30 min dark acclimated plants as in b, exposed to 5 min of low light (LL: 90 µmol photons m^{-2} s^{-1}), 5 min high light (HL: 900 µmol photons m^{-2} s^{-1}), 5 min LL and 5 min darkness (D). **e**, Immunoblot analysis of thylakoid (T) and soluble (S) fractions from isolated thylakoids incubated with buffer alone (-), 2 M NaBr, 0.1 M Na_2CO_3 or 2 M NaSCN from plants as indicated below the blotting results using a KEA3 specific antibody. **f**, Model explaining results in e. In the presence of KEA3 in the thylakoid membrane stromal RCT is stabilized via protein-protein interactions. In the absence of KEA3, RCT has some capacity to interact with the thylakoid membrane, but is rapidly washed off with buffer (RCT, regulatory C-terminus of KEA3; S, stroma and L, lumen).

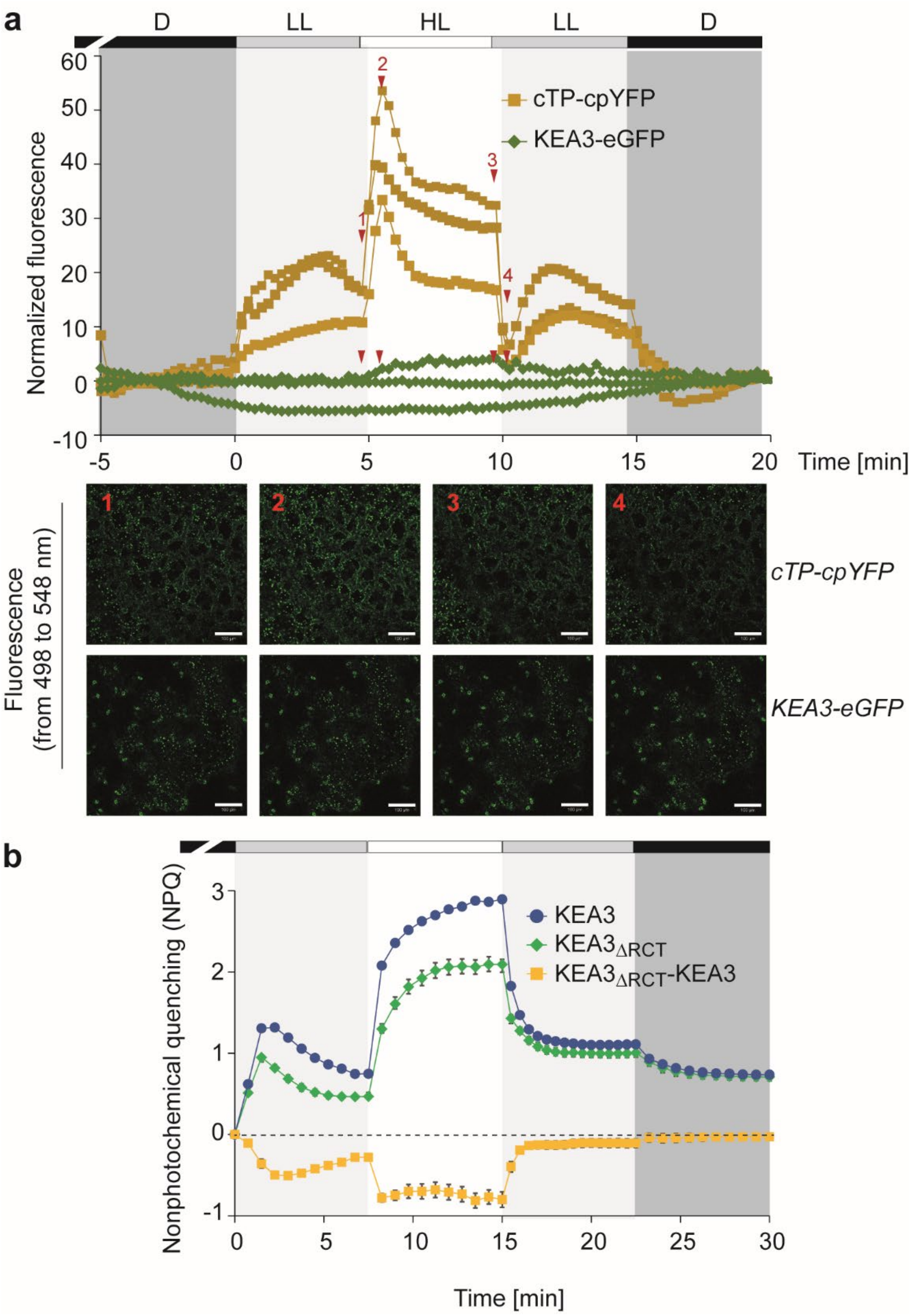

Supplementary Fig. 4. pH transients and KEA3 regulation coincide. a, Three independent baseline-subtracted fluorescence recordings of plants harboring either a chloroplast targeted cpYFP or KEA3-eGFP used to generate the graph shown in Fig. 2a (D, darkness; LL, low light of 90 µmol photons m^{-2} s^{-1}; HL, high light of 900 µmol photons m^{-2} s^{-1}). Numbered arrows in red indicate time points at which representative microscopy pictures are displayed below the graph. Scale bars corresponds to 100 µM. **b**, Plants expressing WT-levels of KEA3 and $KEA3_{\Delta RCT}$ (KEA3-eGFP in *kea3-1* and $KEA3_{\Delta RCT}$-eGFP in *kea3-1*, respectively) were exposed to the same fluctuating light treatment as in a and NPQ was calculated from Chl *a* fluorescence. The NPQ difference between $KEA3_{\Delta RCT}$ and KEA3 was calculated and reflects the loss of NPQ in $KEA3_{\Delta RCT}$ due to the absence of proton antiport regulation via the RCT. Average is shown for n = 5 ± SD of biological replicates.

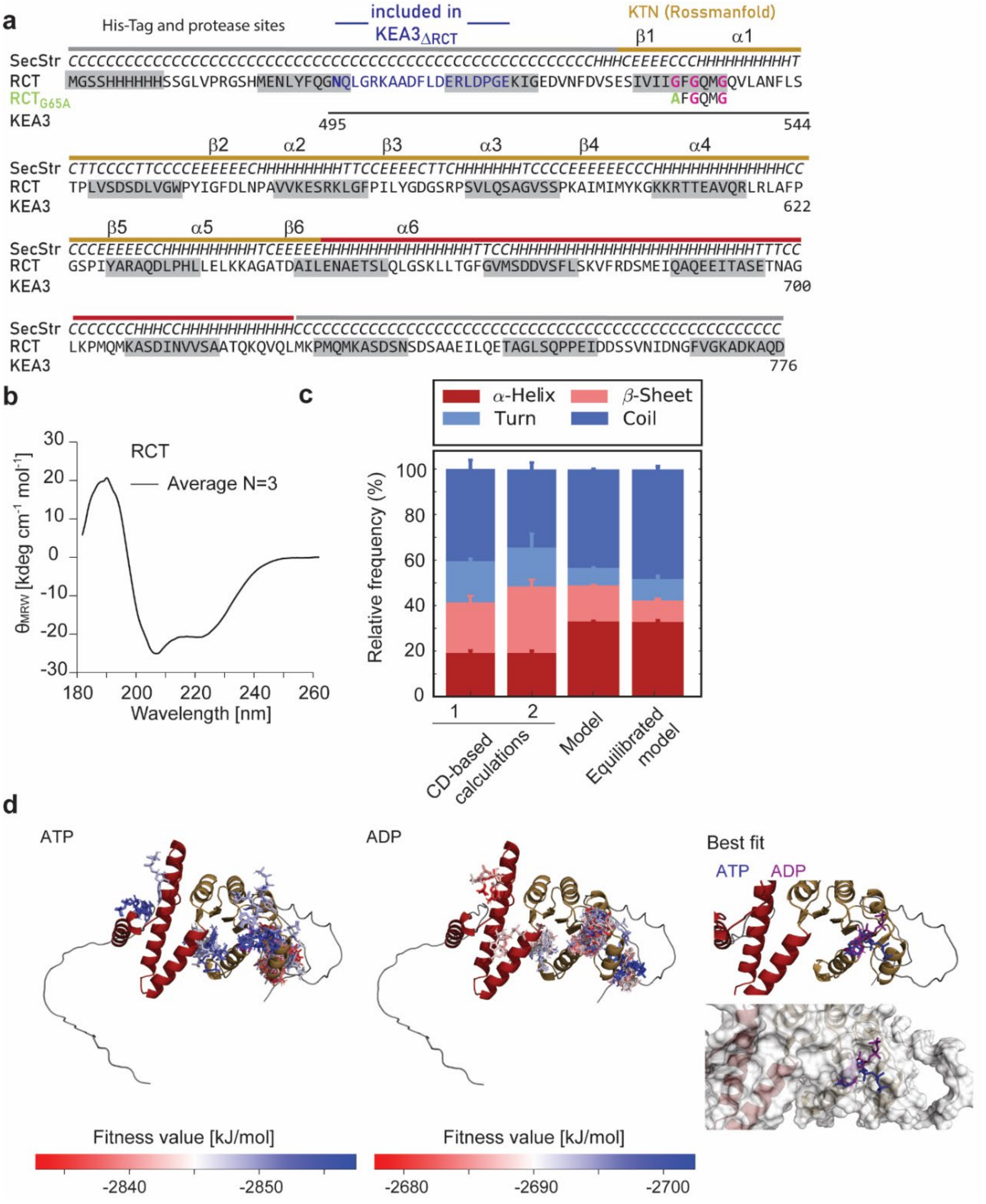

Supplementary Fig. 5. RCT sequence and structure, experimental verification and ATP docking. **a,** Sequence, predicted secondary structure and folds of the recombinant RCT with its N-terminal His-tag. Blue highlights the sequence retained in KEA3$_{\Delta RCT}$ and start of RCT. The two conserved glycine stretches are marked in purple, the alanine replacement at G65 in green. The RCT spans KEA3 residues 495-776 (SecStr, secondary structure: C, coil; T, turn; H, α-helix; E, β -sheet, β1-6 and α1-6 denote the secondary structure elements of the Rossmann fold). **b,** Far-UV spectrum of recombinant RCT used for calculating the secondary structure composition[1]. Average of n=3 technical replicates is shown. **c**, Secondary structure composition as estimated from the experimental data in (b) according to reference spectra of native globular proteins (1) and denatured proteins (2) with n = 3, the model built by AlphaFold2 and the equilibrated model after 1 µs molecular dynamics simulation with n = 10 simulations. Error bars indicate SD. **d**, The RCT model was used for *in silico* docking experiments using SwissDock and applying 256 different ATP or ADP conformations, respectively. Docking results are ranked according to SwissDock's fitness value with blue indicating the strongest interaction. The lowest fitness value (best fit) for ADP and ATP docking was found with the adenosine moiety in the same binding pocket close to G65.

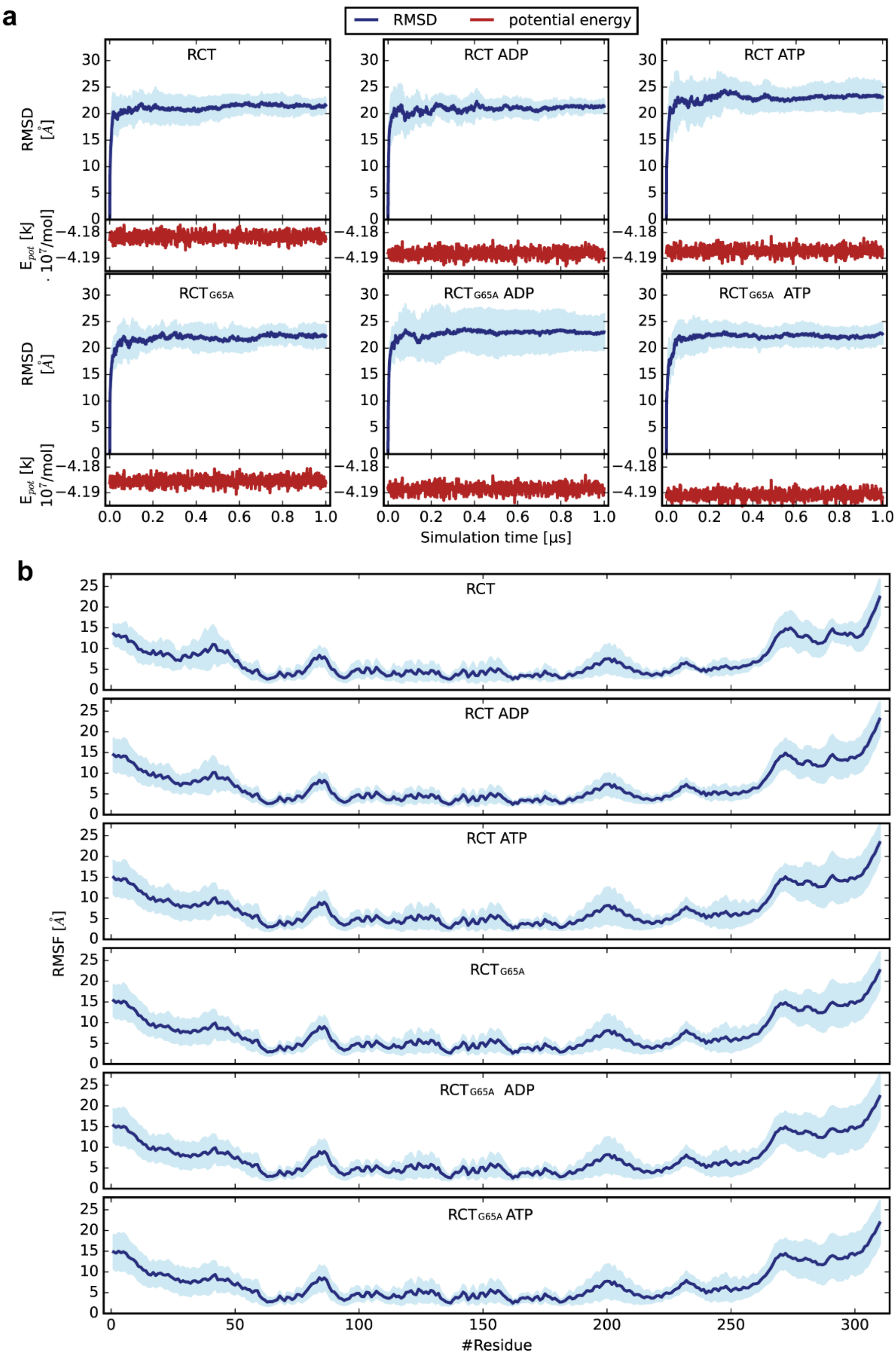

Supplementary Fig. 6 RMSD, potential energy and RMSF during MD simulations. a, The upper panel in each subplot represents the development of the root mean square deviation (RMSD in Å, averages of n=10 ± SD) based on the backbone atoms of the RCT (top) and RCT_{G65A} (bottom) for MD systems alone or with the ligands ADP or ATP bound to the site determined by *in-silico* docking over the simulation time. The lower panel indicates the averaged potential energy for the corresponding simulation system in the upper subplot. The standard deviations (n=10) are marked in lighter colors. **b**, The root mean square fluctuation (RMSF) in Å of each amino acid residue along the protein sequence during the simulations is depicted for respective systems in **a**.

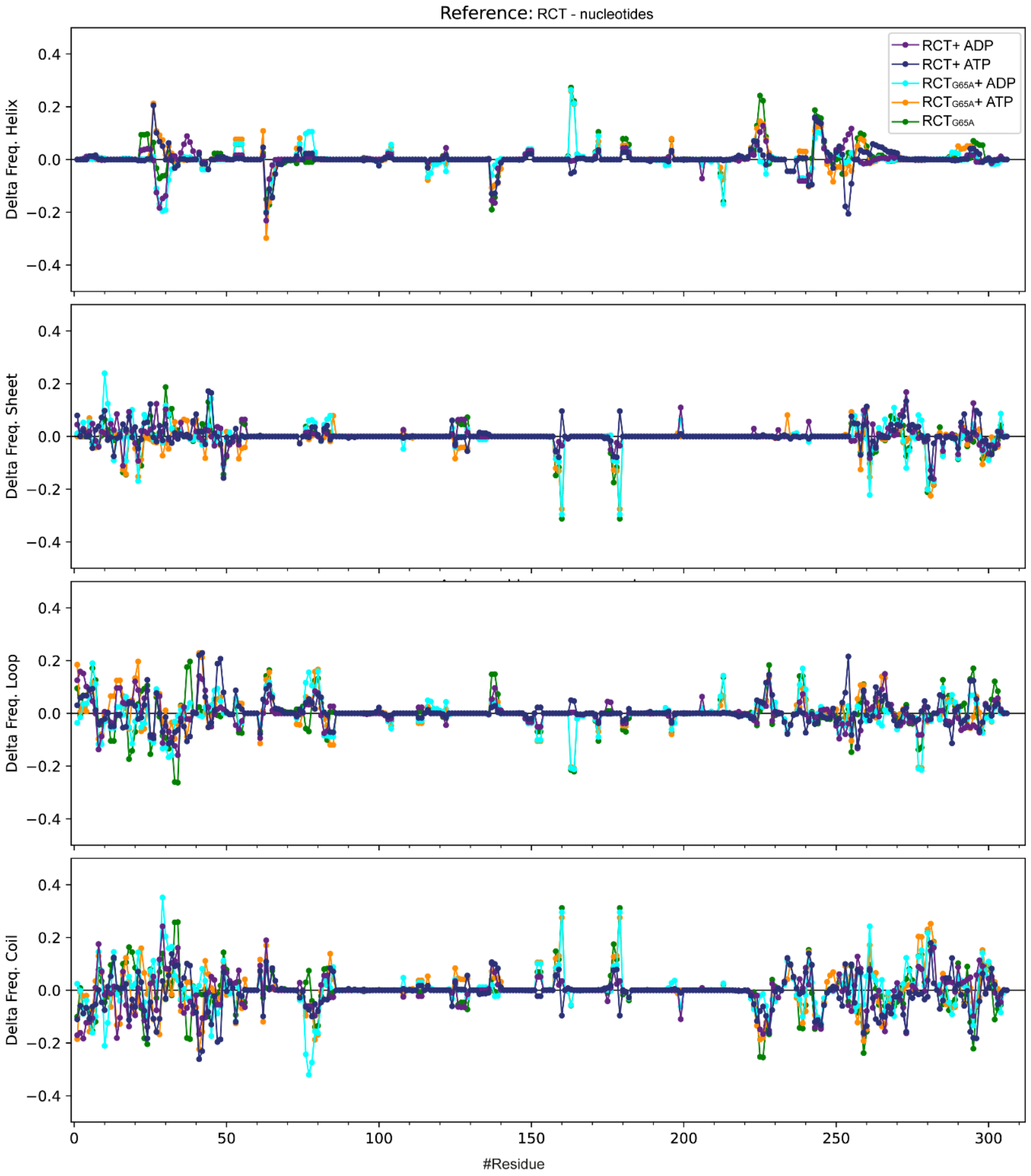

Supplementary Fig. 7. Differences in secondary structure elements to RCT without nucleotides. Frequencies of the secondary structure elements α-helix, ß-sheet, loop and coil along the RCT or RCT_{G65A} protein sequence of the RCT (ADP, ATP) and the RCT_{G65A} systems (without nucleotides, ADP, ATP) are displayed as the difference to ligand-free RCT. Frequencies are averaged over n = 10 simulations.

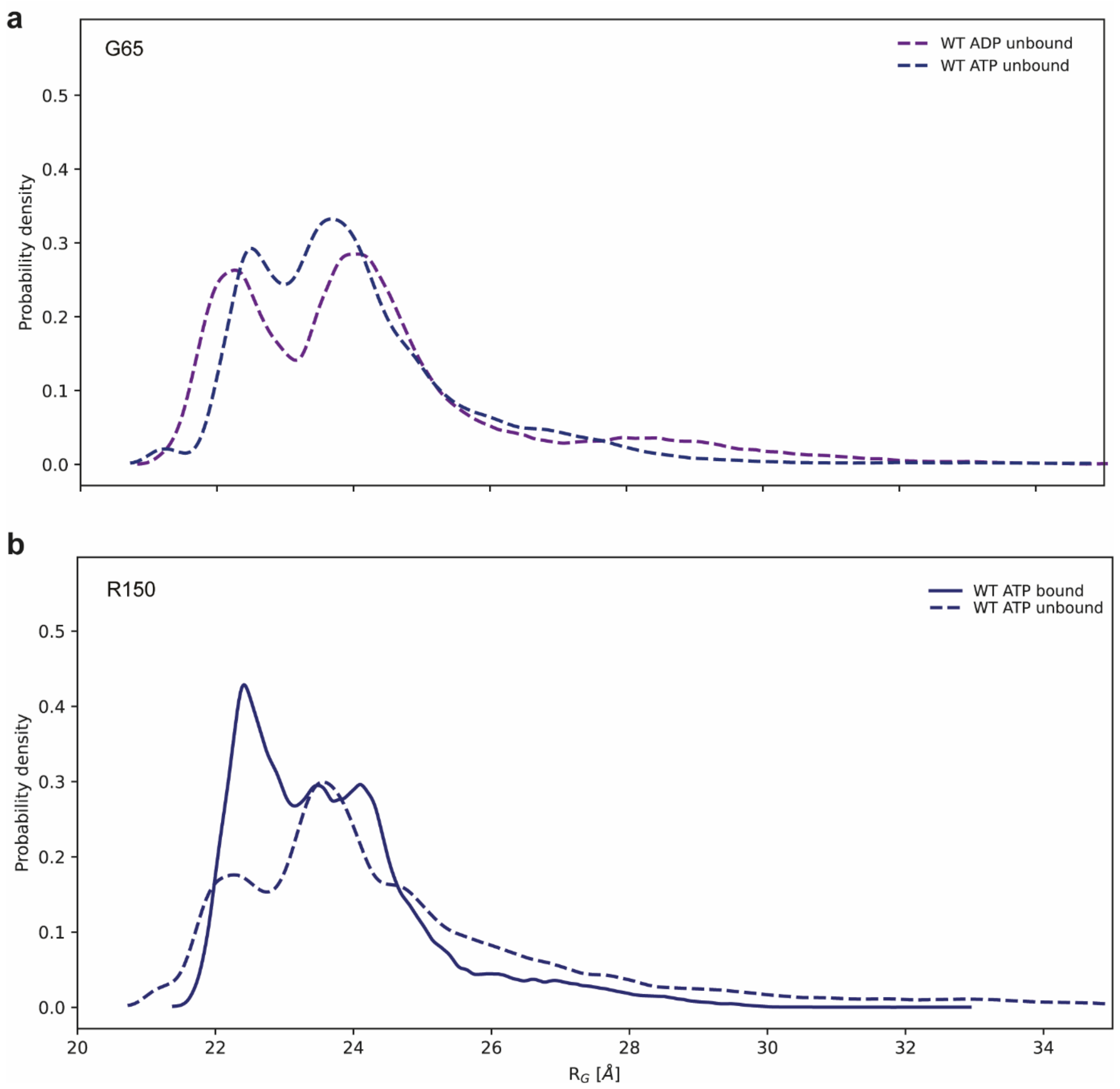

Supplementary Fig. 8 Binding frequency of ATP with the residues G65 and R150 of RCT. a-b, The probability density for radii of gyration from the MD simulation of RCT when ATP and ADP were not bound to G65 (**a**) and ATP is bound and not bound to R150 (**b**). Binding is defined as distance of nucleotides to residue < 7 Å.

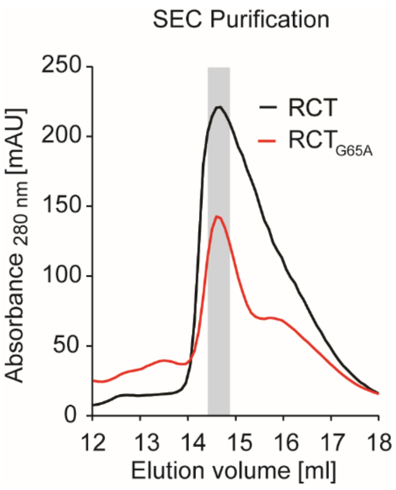

Supplementary Fig. 9 Recombinant RCT purification. Recombinant RCT and RCT_{G65A} were purified via size exclusion chromatography and eluted at the same elution volume of 14.5 – 15.0 ml for subsequent nucleotide binding analyses.

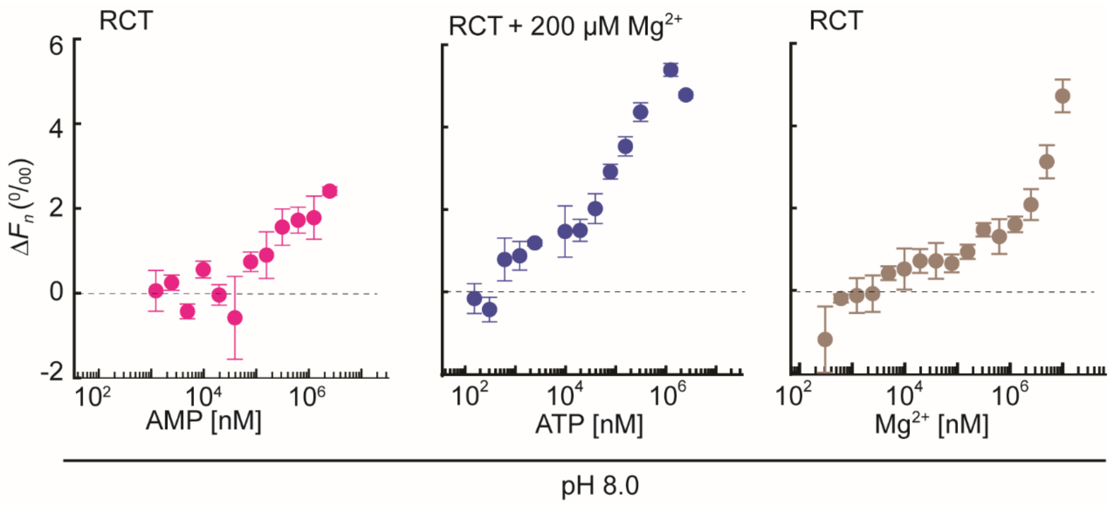

Supplementary Fig. 10. Additional MST analyses of the RCT at pH 8.0 to test AMP and effect of Mg^{2+} on ATP binding. n = 3 ± SD of technical replicates are shown

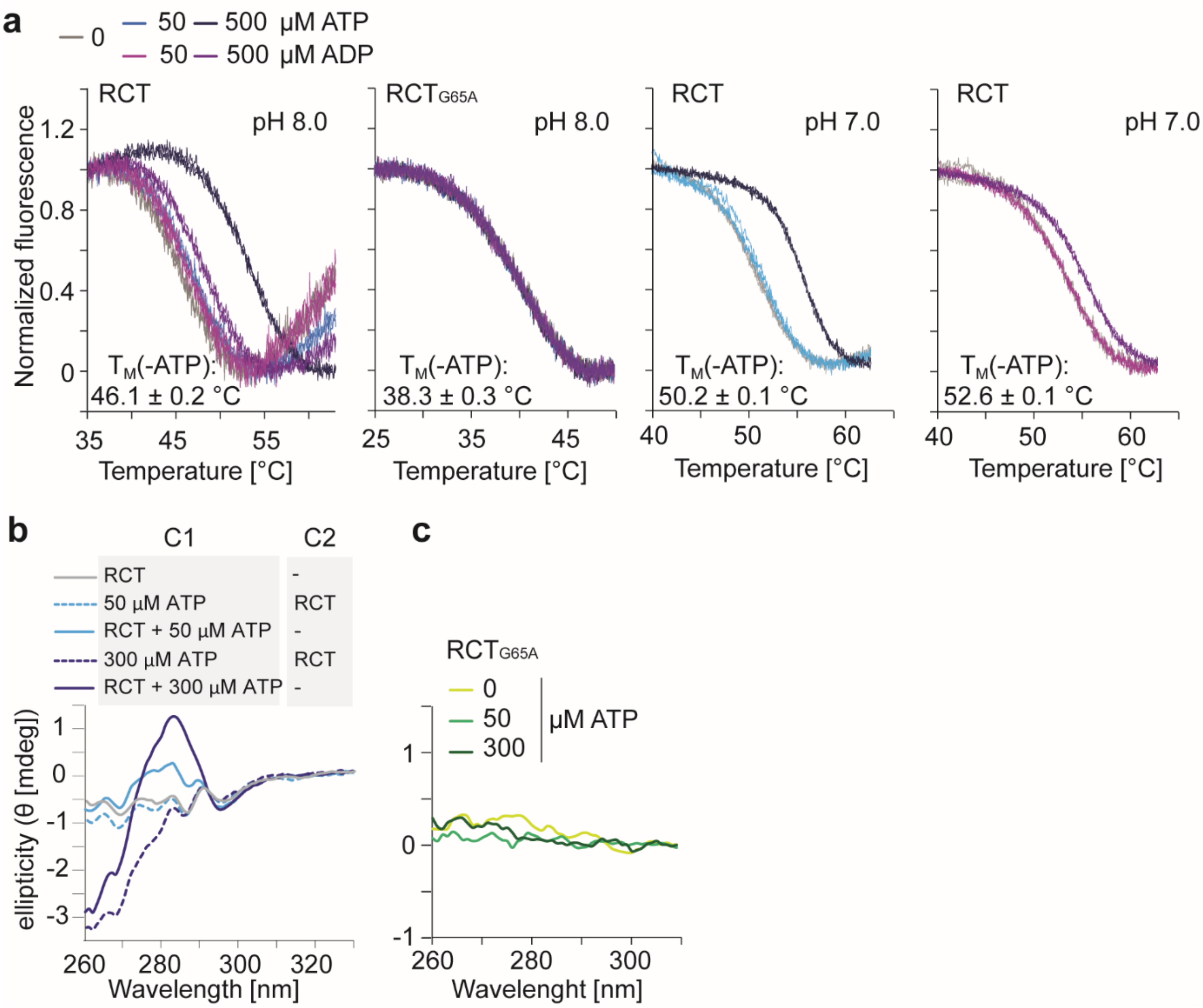

Supplementary Fig. 11 Upon ATP binding the RCT undergoes a conformational change, which results in increased thermal stability and differences in CD spectra. a, Differential scanning fluorimetry was used to record melting curves for analyzing effects of ATP or ADP binding on the thermal stability of RCT and RCT_{G65A} at pH 7.0 and pH 8.0. Single traces for n = 3 technical replicates are shown. The melting temperature (T_M) was derived from the minimum of the first derivative of the melting curve. **b**, Near-UV CD spectra were recorded with a two-cuvette set-up (C, cuvette) and revealed that RCT undergoes a conformational change in response to ATP binding at pH 8. **c**, RCT_{G65A} was measured at pH 8.0 with different concentrations of ATP, which did not greatly affect ellipticity.

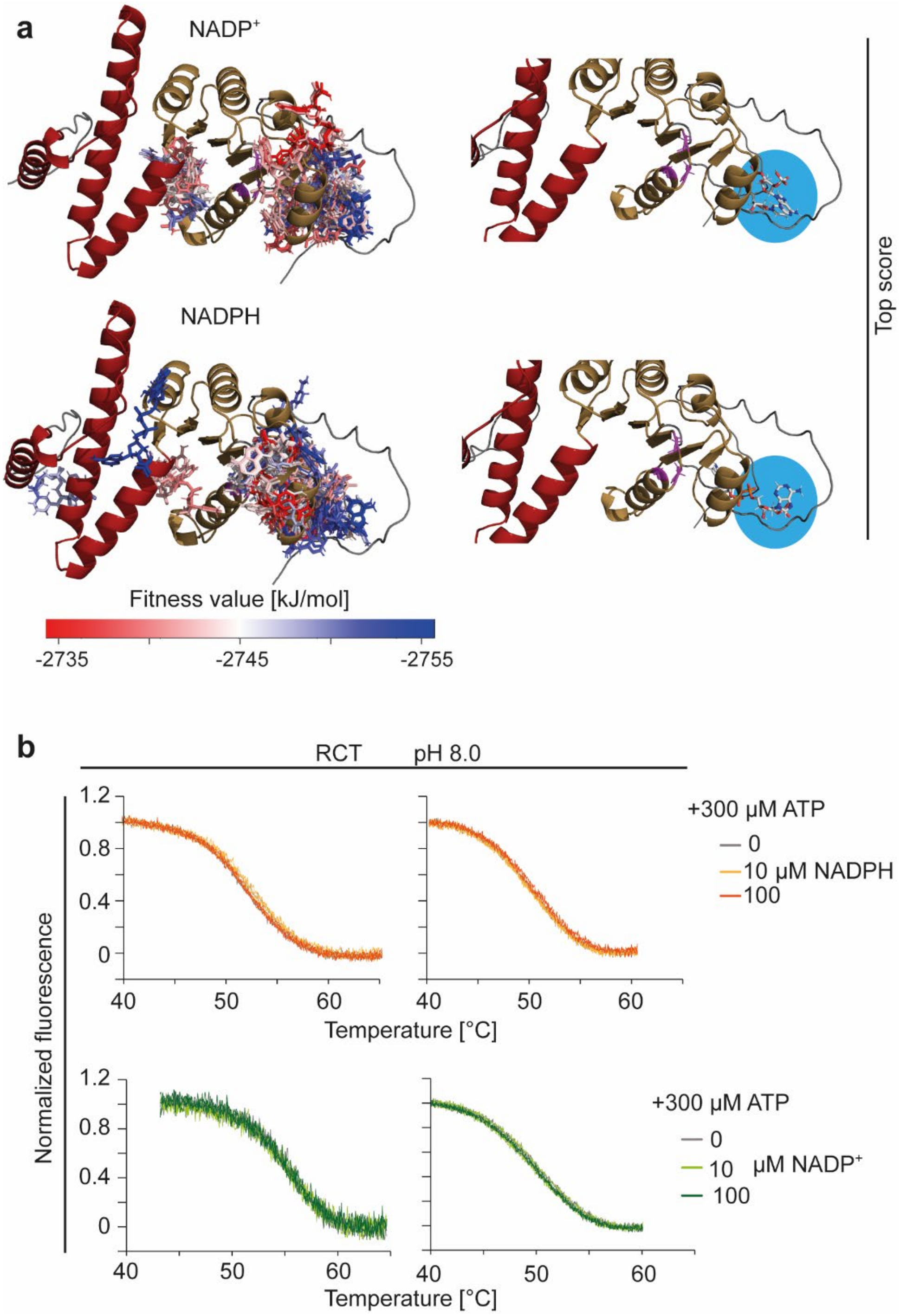

Supplementary Fig. 12 NADPH and NADP⁺ do not dock to the KTN ATP, ADP binding site and have no effect on thermal stability of the RCT. a, The RCT model was used for *in silico* docking experiments using SwissDock and applying 256 different NADP+ or NADPH conformations, respectively. Docking results are ranked according to SwissDock's fitness value with blue indicating the strongest interaction. The lowest fitness value (best fit) was found close to α-helix 2 of the Rossmann fold, not in the binding pocket of ATP and ADP (shown on the right). The blue circle highlights the best fit position for each nucleotide. **b**, Differential scanning fluorimetry was used to record melting curves for analyzing effects of NADPH or NADP⁺ on the thermal stability of RCT at pH 8.0 with and without saturating concentrations of ATP. Single traces for n = 3 technical replicates are shown. Neither NADPH nor NADP⁺ appeared to influence thermostability under the analyzed conditions.

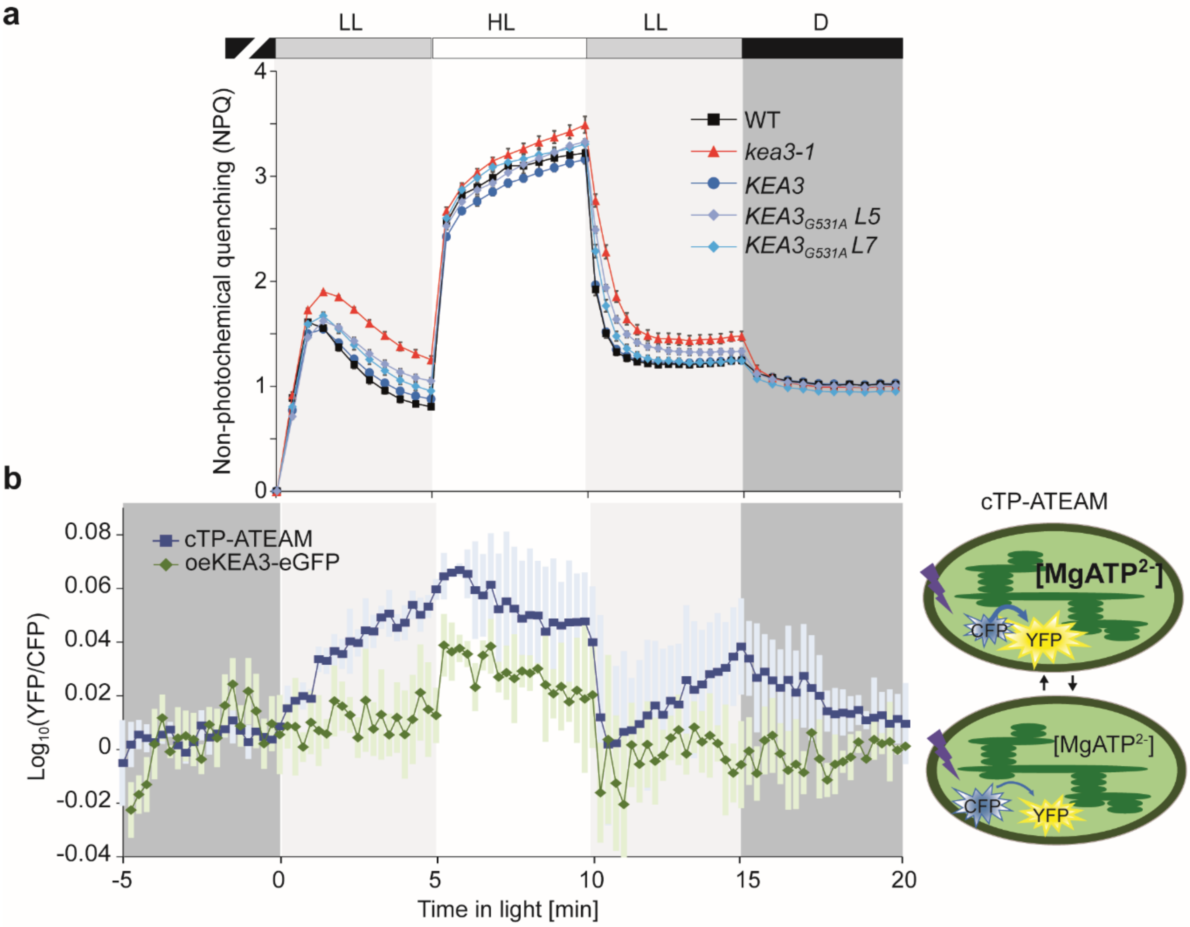

Supplementary Fig. 13 NPQ of different lines and MgATP^{2-} measurements during changes in light intensity. a, The different Arabidopsis lines shown in Fig. 8 were dark acclimated for 30 min and then exposed to an alternating light regime of 5 min low light (LL, 90 µmol photons m^{-2} s^{-1}), 5 min high light (HL, 900 µmol photons m^{-2} s^{-1}), 5 min LL and 5 min darkness and non-photochemical quenching (NPQ) was determined from chlorophyll a fluorescence analyses. n = 3 (WT, *kea3-1*), n = 12 (KEA3, *KEA3$_{G531A}$* L5 and L7) biological replicates. Error bars = SE. Single data points are plotted in the Source data file. **b**, Leaf discs of cTP-ATeam or oeKEA3-eGFP (control) were dark-acclimated (D, darkness) and examined by confocal microscopy using a light box, which supplied red light between 620 and 645 nm wavelengths at two different light intensities (low light, LL, 90, and high light, HL, 900 µmol photons m^{-2} s^{-1}) during indicated time intervals. Excitation was provided at 458 nm and fluorescence was collected for CFP at 470 – 507 nm and for YFP at 521 – 531 nm. The fluorescence signals for YFP/CFP were log10- transformed and a baseline from the last minute of each dark phase (before and after the light treatment) was subtracted. Average is shown for biological replicates, n = 3 ± SD. Note that also the YFP/CFP ratio of the control shows some reaction to changes in light intensity.

Supplementary Table 1: Results of Two Way ANOVA on NPQ during high to low light transition as in Fig. 8.

Two Way Analysis of Variance
General Linear Model
Dependent Variable: NPQ

Normality Test (Shapiro-Wilk):	Passed	(P = 0.312)
Equal Variance Test (Brown-Forsythe):	Passed	(P = 0.797)

Source of Variation	DF	SS	MS	F	P
Genotype	4	6.342	1.586	114.883	<0.001
Time	4	75.631	18.908	1370.021	<0.001
Genotype x Time	16	1.078	0.0674	4.883	<0.001
Residual	185	2.553	0.0138		
Total	209	116.777	0.559		

Main effects cannot be properly interpreted if significant interaction is determined. This is because the size of a factor's effect depends upon the level of the other factor.
The effect of different levels of Genotype depends on what level of Time is present. There is a statistically significant interaction between Genotype and Time. (P = <0.001)

Power of performed test with alpha = 0.0500: for Genotype : 1.000
Power of performed test with alpha = 0.0500: for Time : 1.000
Power of performed test with alpha = 0.0500: for Genotype x Time : 1.000

All Pairwise Multiple Comparison Procedures
(Holm-Sidak method):

Overall significance level = 0.05
Comparisons for factor: Genotype

Comparison	**Diff of Means**	**t**	**P**	**P<0.050**
kea3 vs. *KEA3*	0.544	17.929	<0.001	Yes
L5 vs. *KEA3*	0.335	15.268	<0.001	Yes
kea3 vs. WT	0.54	13.464	<0.001	Yes
kea3 vs. L7	0.342	11.274	<0.001	Yes
L5 vs. WT	0.331	9.68	<0.001	Yes
L7 vs. KEA3	0.202	9.412	<0.001	Yes
kea3 vs. L5	0.209	6.814	<0.001	Yes
L5 vs. L7	0.133	6.062	<0.001	Yes
L7 vs. WT	0.198	5.848	<0.001	Yes
WT vs. KEA3	0.00357	0.105	0.916	No

Comparisons for factor: Time

0 vs. 80	1.96	64.335	<0.001	Yes
0 vs. 60	1.84	60.38	<0.001	Yes
0 vs. 40	1.563	51.282	<0.001	Yes
0 vs. 20	1.057	34.7	<0.001	Yes
20 vs. 80	0.903	29.635	<0.001	Yes
20 vs. 60	0.782	25.68	<0.001	Yes
20 vs. 40	0.505	16.582	<0.001	Yes
40 vs. 80	0.398	13.053	<0.001	Yes
40 vs. 60	0.277	9.098	<0.001	Yes
60 vs. 80	0.121	3.955	<0.001	Yes

Comparisons for factor: Time within WT

0 vs. 80	2.009	20.942	<0.001	Yes
0 vs. 60	1.951	20.34	<0.001	Yes
0 vs. 40	1.768	18.433	<0.001	Yes
0 vs. 20	1.336	13.923	<0.001	Yes
20 vs. 80	0.673	7.019	<0.001	Yes
20 vs. 60	0.615	6.416	<0.001	Yes
20 vs. 40	0.433	4.51	<0.001	Yes
40 vs. 80	0.241	2.509	0.038	Yes
40 vs. 60	0.183	1.906	0.113	No
60 vs. 80	0.0578	0.603	0.547	No

Supplementary Table 1 cont.

Comparisons for factor: Time within *kea3*				
Comparison	**Diff of Means**	**t**	**P**	**P<0.050**
0 vs. 80	1.937	23.317	<0.001	Yes
0 vs. 60	1.727	20.789	<0.001	Yes
0 vs. 40	1.303	15.691	<0.001	Yes
0 vs. 20	1.162	13.986	<0.001	Yes
20 vs. 80	0.952	11.458	<0.001	Yes
20 vs. 60	0.775	9.331	<0.001	Yes
20 vs. 40	0.633	7.625	<0.001	Yes
40 vs. 80	0.528	6.361	<0.001	Yes
40 vs. 60	0.423	5.098	<0.001	Yes
60 vs. 80	0.21	2.528	0.012	Yes

Comparisons for factor: Time within KEA3				
0 vs. 80	1.945	40.559	<0.001	Yes
0 vs. 60	1.879	39.169	<0.001	Yes
0 vs. 40	1.706	35.579	<0.001	Yes
0 vs. 20	1.251	26.084	<0.001	Yes
20 vs. 80	0.694	14.475	<0.001	Yes
20 vs. 60	0.628	13.084	<0.001	Yes
20 vs. 40	0.455	9.495	<0.001	Yes
40 vs. 80	0.239	4.98	<0.001	Yes
40 vs. 60	0.172	3.589	<0.001	Yes
60 vs. 80	0.0667	1.391	0.166	No

Comparisons for factor: Time within L7				
0 vs. 80	2.012	41.946	<0.001	Yes
0 vs. 60	1.891	39.425	<0.001	Yes
0 vs. 40	1.594	33.236	<0.001	Yes
0 vs. 20	1.056	22.018	<0.001	Yes
20 vs. 80	0.956	19.928	<0.001	Yes
20 vs. 60	0.835	17.407	<0.001	Yes
20 vs. 40	0.538	11.217	<0.001	Yes
40 vs. 80	0.418	8.711	<0.001	Yes
40 vs. 60	0.297	6.189	<0.001	Yes
60 vs. 80	0.121	2.521	0.013	Yes

Comparisons for factor: Time within L5				
0 vs. 80	1.899	37.907	<0.001	Yes
0 vs. 60	1.752	34.968	<0.001	Yes
0 vs. 40	1.441	28.765	<0.001	Yes
0 vs. 20	1.03	20.561	<0.001	Yes
20 vs. 80	0.883	17.622	<0.001	Yes
20 vs. 60	0.869	17.346	<0.001	Yes
20 vs. 40	0.572	11.419	<0.001	Yes
40 vs. 80	0.458	9.142	<0.001	Yes
40 vs. 60	0.311	6.203	<0.001	Yes
60 vs. 80	0.147	2.939	0.004	Yes

Comparisons for factor: Genotype within 0 s				
kea3 vs. KEA3	0.336	4.955	<0.001	Yes
L5 vs. KEA3	0.171	3.48	0.006	Yes
L7 vs. KEA3	0.156	3.256	0.011	Yes
kea3 vs. WT	0.276	3.077	0.017	Yes
kea3 vs. L7	0.18	2.653	0.051	No
kea3 vs. L5	0.165	2.412	0.081	No
L5 vs. WT	0.111	1.446	0.478	No
L7 vs. WT	0.0961	1.268	0.5	No
WT vs. KEA3	0.06	0.792	0.675	No
L5 vs. L7	0.0145	0.296	0.768	No

Supplementary Table 1 cont.

Comparisons for factor: Genotype within 20 s

Comparison	Diff of Means	t	P	P<0.050
kea3 vs. KEA3	0.812	11.972	<0.001	Yes
L5 vs. KEA3	0.553	11.272	<0.001	Yes
kea3 vs. WT	0.836	9.323	<0.001	Yes
L5 vs. WT	0.577	7.544	<0.001	Yes
L7 vs. KEA3	0.351	7.322	<0.001	Yes
kea3 vs. L7	0.461	6.794	<0.001	Yes
L7 vs. WT	0.376	4.954	<0.001	Yes
L5 vs. L7	0.202	4.111	<0.001	Yes
kea3 vs. L5	0.259	3.779	<0.001	Yes
KEA3 vs. WT	0.0245	0.323	0.747	No

Comparisons for factor: Genotype within 40 s

kea3 vs. KEA3	0.739	10.896	<0.001	Yes
L5 vs. KEA3	0.436	8.894	<0.001	Yes
kea3 vs. WT	0.741	8.256	<0.001	Yes
kea3 vs. L7	0.47	6.936	<0.001	Yes
L5 vs. WT	0.438	5.722	<0.001	Yes
L7 vs. KEA3	0.269	5.6	<0.001	Yes
kea3 vs. L5	0.303	4.416	<0.001	Yes
L7 vs. WT	0.27	3.564	0.001	Yes
L5 vs. L7	0.168	3.417	0.002	Yes
KEA3 vs. WT	0.00173	0.0228	0.982	No

Comparisons for factor: Genotype within 60 s

kea3 vs. KEA3	0.488	7.19	<0.001	Yes
L5 vs. KEA3	0.298	6.068	<0.001	Yes
kea3 vs. WT	0.5	5.574	<0.001	Yes
kea3 vs. L7	0.344	5.069	<0.001	Yes
L5 vs. WT	0.31	4.051	<0.001	Yes
L5 vs. L7	0.154	3.134	0.01	Yes
L7 vs. KEA3	0.144	3	0.012	Yes
kea3 vs. L5	0.19	2.772	0.018	Yes
L7 vs. WT	0.156	2.061	0.08	No
KEA3 vs. WT	0.0124	0.164	0.87	No

Comparisons for factor: Genotype within 80 s

kea3 vs. KEA3	0.344	5.078	<0.001	Yes
L5 vs. KEA3	0.217	4.426	<0.001	Yes
kea3 vs. WT	0.348	3.878	0.001	Yes
kea3 vs. L7	0.255	3.756	0.002	Yes
L5 vs. WT	0.221	2.883	0.026	Yes
L5 vs. L7	0.127	2.598	0.05	Yes
L7 vs. KEA3	0.0896	1.869	0.23	No
kea3 vs. L5	0.127	1.857	0.23	No
L7 vs. WT	0.0932	1.229	0.393	No
KEA3 vs. WT	0.00353	0.0466	0.963	No

Supplementary Table 2 Vectors generated for self-assembling GFP analysis

Coding sequences of *KEA3*, *cpSRP43*, ALB3 or the transit sequence of the small rubisco subunit (TSrbcs) were amplified from cDNA and inserted into *Kpn*I and *Spe*I restriction sites of pAVA-saGFP1-10, pAVA-saGFP11N or pAVA-saGFP11C resulting in saGFP1-10 (large GFP fragment; saGFP1) or N- or C-terminal saGFP11 (small GFP fragment; saGFP2) fusions, respectively[2].
The plasmid pAVA-saGFP11N was further modified introducing two additional restriction sites (*Bgl*II and *Xma*JI) upstream of saGFP11 using site directed mutagenesis (SDM). These restriction sites were used to introduce the transit peptide of the RubisCo small subunit (TSrbcs; *At5g38410*) leading to the plasmid pAVA-TSrbcs-saGFP11N. Mature KEA3 was introduced into this plasmid downstream of saGFP11 resulting in a TSrbcs-saGFP11-KEA3 fusion (saGFP2 N-KEA). Additionally, precursor KEA3 containing the chloroplast targeting peptide was cloned into pAVA-saGFP11C resulting in a C-terminally fused saGFP11 (saGFP2 KEA-C). As a control, precursor *ALB3* cDNA was cloned into pAVA-saGFP11C (saGFP2 Alb3-C). The chloroplast localization of the large saGFP1-10 fragment was ensured either by C-terminal fusion to TSrbcs (saGFP1 Str) or to TSrbcs-cpSRP43 (saGFP1 Str-SRP43-C). TSrbcs was inserted via in-fusion cloning according to manufacturer's instructions (Takara) into the *Kpn*I linearized pAVA-saGFP1-10 plasmid. TSrbcs-cpSRP43 was introduced into *Kpn*I and *Spe*I restriction sites of pAVA-saGFP1-10.

#	Plasmid	Information	Primer sequences used (5' → 3')
1	pAVA-saGFP11N*	To obtain N-terminal saGFP2 fusions	
2	pAVA-saGFP11N BglII	*Bgl*II site introduction into #1 via SDM	GCAGCAATTTAAATCAGATCTTTTAAAGCAAAAGC<br>GCTTTTGCTTTAAAAGATCTGATTTAAATTGCTGC
3	pAVA-saGFP11N BglII, XmaJI	*Xma*JI site introduction into #2 via SDM	GCAATTTTCTGAAAATTTTCACCTAGGACGAACGATAGCCATGCG<br>CGCATGGCTATCGTTCGTCCTAGGTGAAAATTTTCAGAAAATTGC
4	pAVA-TSrbcs-saGFP11N	TSrbcs introduction into #3	TAGAGATCTATGGCTTCTATGATA<br>CTACCTAGGTACTTTACTCTTCCACC
5	pAVA-TSrbcs-saGFP11-KEA3	Introduction of mature *KEA3* into #4 to obtain saGFP2-N-KEA3	s. Supplementary Table 3
6	pAVA-saGFP11C*	To obtain C-terminal saGFP2 fusions	
7	pAVA-KEA3-saGFP11	Introduction of full length *KEA3* into #6 to obtain KEA3-C-saGFP2	s. Supplementary Table 3
8	pAVA-Alb3-saGFP11	Introduction of full length *Alb3* into #6	GCTGGTACCATGGCGAGAGTTCTA<br>AGCACTAGTTACAGTGCGTTTCCG
9	pAVA-saGFP1-10*	For cytosolic saGFP1	
10	pAVA-TSrbcs-saGFP1-10	For stromal saGFP1	GCAGCAATTTAAATCAGATCTATGGCTTCTATGATA<br>TAGTCATGCGGCCGCGGTACCACTTTACTCTTCCAC
11	pAVA-TSrbcs-cpSRP43-saGFP1-10	Introduction of full length *cpSRP43* into #10 to obtain cpSRP43-C-saGFP1	TAGGGTACCATGGCTTCTATGATA<br>CTAACTAGTTTCATTCATTGGTTGTTGTTG

* kindly provided by E. Schleiff (Frankfurt, Germany), containing sequences provided by G. S. Waldo (Los Alamos, NM, USA)[3]

Supplementary Table 3: Further primer sequences used in this study.

Primer sequences (5' → 3')	Purpose
TGGCACAGGTTCTGGCCAATTTTTTGTCAACGC	Site-directed mutagenesis to derive RCT_{G65A}
TTTGTGCAAATGCAATGATGACAATTGATTCACTAAC	
TTGGCATGGGACTAACTCAG	Genotyping of $KEA3_{G531A}$ plants
CACAATCAGACCACCCAAGA	
TCATTTGGAGAGAACACGGGGGACTCTAGATGGCTTCTATGATATCCTCTTC	Amplification of TSrbcs (*At5g38410*) for stromal RCT overexpression using the *pGWB2* vector
TTCCAAGTTGGTTCTTTACTCTTCCACCATTGC	
TGGAAGAGTAAAGAACCAACTTGGAAGAAAAG	Amplification of RCT with C-terminal Myc-tag for overexpression using the *pGWB2* vector
TTGAACGATCGGGGAAATTCGAGCTCTTACAGATCCTCTTCTGAGATGAGTTTTTGTTCATCTTGAGCTTTATCAGCTTTAC	
ATCAAGCATTCTACGGGTACCATGGCAATTAGTACTATGTTAG	Amplification of the full length *KEA3* coding sequence without stop codon for insertion upstream of the saGFP2 coding sequence in the pAVA vector for transient expression of KEA3-C-saGFP2.
CAGACCCTCCATCGGATCCACTAGTTGATATCACCACTTTGTAC	
CAATCACCGGATTGGGGTACCATGGCAATTAGTACTATGTTAG	Amplification of the *KEA3* coding sequence with stop codon for insertion downstream of TSrbcs and the *saGFP2* coding sequence in the pAVA vector for transient expression of TSrbcs-saGFP2-N-KEA3.
GATTTTTGCGGACTCTAGATTAACTAGTTTAATCTTGAGCTTTATCAGC	
TTGGCATGGGACTAACTCAG	Genotyping of the *kea3-1* mutant (Gabi_170G09)
CACAATCAGACCACCCAAGA	

Supplementary references

1 Sreerama, N. & Woody, R. W. Estimation of protein secondary structure from circular dichroism spectra: comparison of CONTIN, SELCON, and CDSSTR methods with an expanded reference set. *Anal Biochem* **287**, 252-260, doi:10.1006/abio.2000.4880 (2000).

2 Ulrich, T., Gross, L. E., Sommer, M. S., Schleiff, E. & Rapaport, D. Chloroplast β-barrel proteins are assembled into the mitochondrial outer membrane in a process that depends on the TOM and TOB complexes. *J Biol Chem* **287**, 27467-27479, doi:10.1074/jbc.M112.382093 (2012).

3 Cabantous, S., Terwilliger, T. C. & Waldo, G. S. Protein tagging and detection with engineered self-assembling fragments of green fluorescent protein. *Nature Biotechnology* **23**, 102-107, doi:10.1038/nbt1044 (2005).
